# Twenty Years of Elfin Enumeration: Abundance Patterns of Five Species of *Callophrys* (Lycaenidae) in Central Wisconsin, USA

**DOI:** 10.3390/insects5020332

**Published:** 2014-04-23

**Authors:** Ann B. Swengel, Scott R. Swengel

**Affiliations:** 909 Birch Street, Baraboo, WI 53913, USA

**Keywords:** *Callophrys*, *Callophrys irus*, climate effects, elfin, frosted elfin, long-term monitoring, population trends, reserves and non-reserves, Theclinae, pine barrens

## Abstract

We recorded five species of elfins (*Callophrys*) during annual spring surveys targeting frosted elfin *C. irus* (state-listed as threatened) in 19 pine-oak barrens in central Wisconsin USA during 1994–2013. At the northwest end of its range here, *C. irus* co-varied with spring temperature, but declined significantly over time (eight sites verified extant of originally 17). Two other specialists increased significantly. The northern specialist, hoary elfin *C. polios* (nine sites), correlated positively with the previous year’s growing season precipitation. The southern specialist, Henry’s elfin *C. henrici* (11 sites), co-varied with winter precipitation and spring temperature and dryness. The two resident generalists had stable trends. For all species, the first observed date per year became earlier over time and varied more than the last observed date. Thus, flight period span increased with earlier first observed dates. Elfin abundance increased significantly with earlier first observed dates in the current and/or prior year. Three species (*C. irus*, *C. henrici*, a generalist) had more positive population trends in reserves than non-reserves. This suggests that *C. irus* declines correspond to habitat conditions. Thus, monitoring programs and habitat management specifically for *C. irus* appear necessary to obtain a long-term stable trend for this species in Wisconsin.

## 1. Introduction

Surveying and monitoring are necessary components of conservation programs, to identify those species that do and do not require conservation action, to analyze sources of variation, and to monitor the efficacy of conservation actions [1,2,3]. Butterfly abundance differs greatly among generations attributable to climatic variation [1,2] and less often documented in response to parasitoid predation [3]. As a result, long-term monitoring is necessary to assess a butterfly species' status and range of variation, so as to distinguish trends from that background variation [4]. 

In this paper, we analyze twenty-year time series of abundance for five species of co-occurring elfins *Callophrys* in pine-oak barrens in central Wisconsin, USA. Frosted elfin *C. irus* is of conservation concern both in Wisconsin, where it is state-listed as threatened [5], and elsewhere in its range [6,7,8,9,10,11,12,13]. We correlate elfin abundance with phenology (first observed date that year) and to climatic variables and describe patterns of fluctuation among years and compare trend (correlations with year) to degree of conservation effort in the sites and climatic affiliation (southern or northern range relative to Wisconsin). These results should be useful for understanding those elfin species of conservation concern more effectively, for evaluating butterfly conservation methods and for assessing how climatic variation might affect elfin populations. 

## 2. Methods

### 2.1. Sites and Surveys

We conducted butterfly transect surveys along like routes on each visit to each site, similar to Pollard [14], as described in Swengel [15,16], Swengel and Swengel [17,18], and Swengel *et al.* [19]. Walking at a slow pace (2–3 km/h) on parallel routes 5–10 m apart, we counted all adult butterflies observed ahead and to the sides—to the limit an individual could be identified, possibly with the aid of binoculars after detection—and tracked them. Surveys occurred during a wide range of times of day and weather, occasionally in intermittent light drizzle, so long as butterfly activity was apparent, but not in continuous rain. We identified likely larval host plants based on published reports [6,20] cross-referenced to the flora we observed in the study sites. Based on our survey results [15,16,17] and likely host plant associations, we classified a butterfly species as a generalist if it occurred widely in a variety of vegetation types and as a specialist if it was localized in pine-oak barrens in the study region. Per published ranges [20,21,22], we classified the butterflies as northern or southern species based on whether more of their range occurred north or south of Wisconsin. 

**Figure 1:**
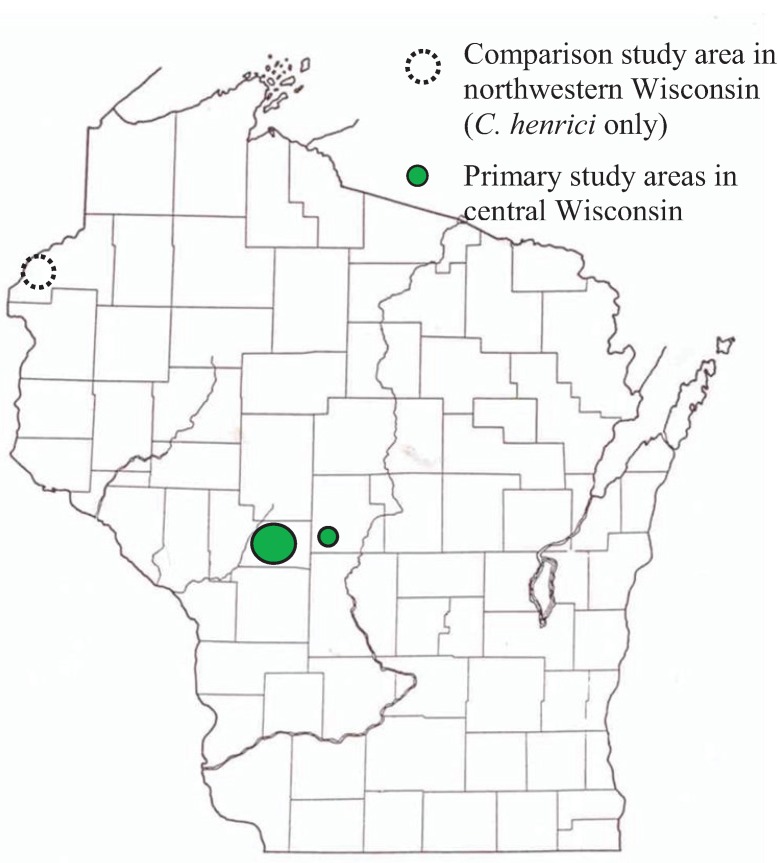
Map showing study regions in central and northwestern Wisconsin.

The study sites are pine-oak barrens in central and northwestern Wisconsin (Figure 1) [23], which have herbaceous flora similar to sand prairies (“sand barrens” in Curtis [24]). Survey dates and locations were selected to study focal specialist species [15,18,25]: *C. irus* (listed in Wisconsin as threatened) and ‘Karner’ Melissa blue *Lycaeides melissa samuelis* (federally listed as endangered) [5,26]. All five species of elfins in this study (Table 1) have similar adult timing, which overlaps with the first part of spring *L. melissa samuelis* adults [18]. The only known host in the wild for *L. melissa samuelis* is wild lupine *Lupinus perennis* [26,27]. This is also the presumed only host for *C. irus* in Wisconsin [15,23] because of the strong association of *C. irus* adults with *L. perennis* and paucity or absence of the alternate known host (*Baptisia*) in these *C. irus* sites in Wisconsin. We made repeated visits to the central Wisconsin study region during 1992–2013 that covered the elfin flight period each year, with timing based on prior visits each year before elfins were seen both in and immediately south of the study region to verify that year’s seasonal progression. 

**Table 1:** Number of long-term sites, sum of peak annual counts of elfin individuals from those sites used in abundance analyses, and likely larval host plants.

Species ^1^	Sites	Individuals	Likely host plant in Wisconsin
G Brown elfin *Callophrys augustinus*	16	111	heaths (Ericaceae)
S Hoary elfin *Callophrys polios*	9	185	bearberry *Arctostaphylos uva-ursi*
G Eastern pine elfin *Callophrys niphon*	15	633	pines *Pinus*
S Henry’s elfin *Callophrys henrici*	11	63	blueberries *Vaccinium*
S Frosted elfin *Callophrys irus*	17	286	wild lupine *Lupinus perennis*

^1^ G = generalist (occurring widely in a variety of vegetation types); S = specialist (localized primarily to barrens).

Study sites were deliberately selected for their conservation interest, *i.e.*, those known or thought to have specialist butterflies. They included conservation lands, forest reserves (some burned by wildfire prior to the study period), and rights-of-way for highways and power lines. It was not possible to visit all sites (>125) every year, but most were visited more than once both within and among years, and a subset of central Wisconsin sites in most or all years. Regulation for the federally listed *L. melissa samuelis* allows two tiers of protective effort designed specifically for this butterfly [25,26]: reserve, where recovery would be expected to occur; and “shifting mosaic” and “permanency of habitat”, where land uses are not required to aim for recovery but must be activities “with consideration for the Karner blue.” All sites analyzed for butterfly abundance in this study have supported *L. melissa samuelis* and are covered by this federal regulation in some manner. 

For each species, our population abundance index is the peak survey count along the same route per site per year. We standardized this to survey time to create an observation rate (relative abundance) per hour per site. One survey during the main flight period has been adequate for producing representative indices for comparisons of relative abundance within and among sites [25,28,29]. We assembled time series for 19 sites in central Wisconsin (Jackson and Wood Counties) surveyed each year during 1994–2013 (Figure 1, Table 2). Because *C. irus* is of conservation concern, we also added 1992–1993 data for that species at nine of the sites also covered during those years (Table 2). It was not possible to survey northwestern Wisconsin annually for elfins. But we assembled available data for comparison to central Wisconsin results for the one analyzable specialist elfin (*C. henrici*) found in Burnett County sites (Figure 1). 

**Table 2:** Descriptive statistics on the long-term study sites surveyed each year 1994–2013.

County, site	Type ^1^	Latitude	Longitude	Route Length (km)	Years found ^2^				
Jackson County									
Bauer 2	SM	44.30	90.75	0.60	3	7	11	1	13
Bauer 3	SM	44.30	90.75	0.60	2	4	11	1	13
Brockway 1	R	44.30	90.743	1.00	8	11	11	5	18
Dike 17 ^3^	R	44.31	90.564	1.45	6	5	13	1	2
North Brockway E ^3^	SM	44.32	90.73	0.55	15	17	13	8	12
S Brockway W 1 ^3^	R	44.281	90.742	0.30	1	2	13	2	12
S Brockway W 4 ^3^	R	44.283	90.744	0.40	1	5	15	7	16
Stanton Road Main ^3^	PH	44.23	90.65	1.70	7	0	19	1	4
West Castle Mound 2 ^3^	SM	44.273	90.764	0.40	0	3	4	0	8
West Castle Mound 4 ^3^	SM	44.273	90.766	0.90	0	0	2	0	8
West Castle Mound 5	PH	44.275	90.765	0.40	1	0	0	0	2
Wildcat-Spangler NE ^3^	SM	44.2782	90.678	0.85	2	0	6	0	3
Wildcat-Spangler SE	SM	44.278	90.678	0.40	1	0	5	0	4
Wood County									
Highway X N-S ^3^	PH	44.34	90.13	1.60	8	7	13	1	16
Highway X E-W	PH	44.30	90.13	1.00	1	0	14	0	0
Highway X S	PH	44.32	90.13	0.45	1	0	7	3	7
Sandhill 2	R	44.33	90.13	0.30	0	0	0	0	3
Sandhill 3	R	44.33	90.15	0.30	1	0	0	0	1
Sandhill 7	R	44.33	90.20	0.65	3	0	0	6	0

^1^ R = reserve, PH = permanency of habitat, SM = shifting mosaic. ^2^ N years each elfin was found in each site, presented in species order of Table 1. ^3^ Time series for *C. irus* at these sites also include 1992–1993.

### 2.2. Analysis

All analyses were done with ABstat 7.20 software [30]. All tests were two-tailed, with statistical significance set at *p* < 0.05. Since significant results occurred at a frequency well above that expected due to spurious Type I statistical error, the critical P value was not lowered further, as more Type II errors (biologically meaningful patterns lacking statistical significance) would be created than Type I errors eliminated. All statistical tests in this study were non-parametric because they did not require data to be distributed normally. We used the Spearman rank correlation for all correlations. We calculated trends (correlation of elfin abundance with year) both for all sites and by site type (reserve, shifting mosaic, permanency of habitat). 

We obtained climate data for winter (December to February), spring (March to May), summer (June to August), the growing season (April to September), and year, by subregion, from the Wisconsin State Climatology Office [31]. We correlated elfin abundance with average temperature and total precipitation (available through 2011), and season-long snowfall total (from the prior year’s fall to the following year’s spring, available through 2010) and the Palmer Drought Severity Index (available through 2010), which becomes more positive in floods and more negative in droughts. Jackson County falls in the west central subregion and Wood County in the central subregion. We matched the appropriate subregion’s climate data to each year of each site’s time series and correlated elfin abundance with climate variables for up to one year after the timing of the climate variable.

We identified our first observed date (FOD) for each elfin species each year in any site in central Wisconsin. We also identified our FOD from farther north if it was earlier than our FOD for the species in central Wisconsin: for four years for *C. augustinus* (two years not found in central; two years found later in central), two years for *C. polios* (found later in central those years), two years for *C. niphon* (found later in central), and four years for *C. henrici* (not found in central those years; plus not found in any of these areas in one additional year). We recorded *C. irus* in central Wisconsin in all study years and never in northwestern Wisconsin, which is outside the known range for this species [20,21,22]. FOD was most rigorous for *C. irus* because we only found the species on the first date we checked any *C. irus* site (1997, 2004, 2006, with only one individual found on each of those first dates) in three years. The other elfin species had more years when found on the first survey of the year. We also identified the last observed date for each species each year in central Wisconsin and N days in flight period in central Wisconsin. We correlated FOD with elfin abundance in the long-term survey sites, year (trend over time), N days in flight period, and spring temperature. We analyzed FOD both ways: (1) limited to central Wisconsin surveys and (2) using data from farther north. 

## 3. Results

### 3.1. First Observed Date (FOD)

**Figure 2:**
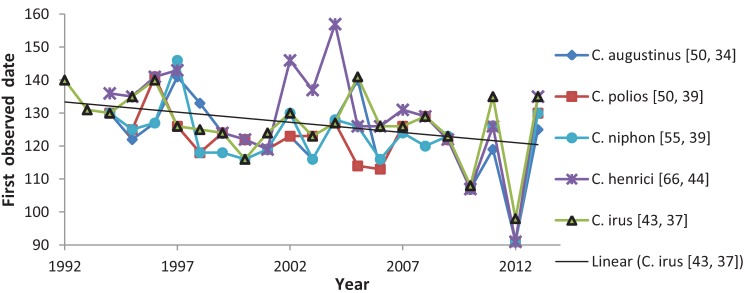
First observed date for each elfin species in central Wisconsin or in 12 instances from farther north if found there earlier than in central Wisconsin. Numbers in parentheses: difference in days between earliest and latest first observed date; difference in days in largest change in first observed date between consecutive years. Y axis: 90 = 31 Mar, 160 = 9 June.

Trend over time in FOD (Figure 2, Table 3) was negative for all species, but this was only significant when testing all species together: Spearman rank correlation r = −0.3047, *p* < 0.01, N = 99 FOD × species × year, for 1994–2013 using all data, including further north, as in Table 3. This correlation was unweighted by abundance or species and had only one missing value (*C. henrici* in 1998). The trend in the last observed date was less consistent and much weaker, with no significant correlations (Table 4). Thus, for all five species, N days in flight period each year correlated negatively with FOD (Table 5), and flight periods were longer when they began earlier, significantly so for each species except *C. polios*. The longest recorded flight period for each species occurred in 2012, the longest found for *C. niphon* (61 days) and *C. irus* (54 days). *C. irus* FOD significantly co-varied with FOD for all other elfins (Table 6). The change in FOD between the prior year and the current year increased during the study, significantly so for all species combined and for *C. irus*, *C. augustinus*, and *C. henrici* (Table 7). The largest change from one year to the next occurred from 2012 to 2013, becoming later (Figure 2) by 34 (*C. augustinus*) to 44 days (*C. henrici*). The second largest change was from 2011 to 2012, becoming earlier by 28–37 days, except for *C. irus* with a tied change from 2012 to 2013 (37 days).

**Table 3:** Spearman rank correlation coefficients (r) of first observed date and year (trend over time), by species, with date calculated either using surveys only in central Wisconsin or using all survey data including from northern Wisconsin and northeastern Minnesota. *p < 0.05, **p < 0.01, ***p < 0.001.

Species	Central Wisconsin only		All data, including further north	
	N	r	N	r
*C. augustinus*	19	−0.4327	20	−0.4369 (*p <* 0.06)
*C. polios*	20	−0.1358	20	−0.2495
*C. niphon*	20	−0.2264	20	−0.2679
*C. henrici*	15	−0.4076	19	−0.4222 (*p <* 0.07)
*C. irus* (1992–2013)	22	−0.31115	--	
1994–2013	20	−0.1872	--	

**Table 4:** Spearman rank correlation coefficients (r) of last observed date in central Wisconsin and year (trend over time), by species. None were statistically significant.

Species	N	r
*C. augustinus*	19	+0.0026
*C. polios*	20	+0.0340
*C. niphon*	20	−0.0768
*C. henrici*	15	+0.1864
*C. irus*	22	−0.1607

**Table 5:** Spearman rank correlation coefficients (r) of N days in flight period and first observed date, calculated either using surveys only in central Wisconsin or using all survey data including from northern Wisconsin and northeastern Minnesota. * *p* < 0.05, ** *p* < 0.01, *** *p* < 0.001.

Species	First observed date, Central Wisconsin only		All data, including further north	
	N	r	N	r
*C. augustinus*	19	−0.7600 **	20	−0.6145 **
*C. polios*	20	−0.2169	20	−0.2497
*C. niphon*	20	−0.4703	20	−0.7237 **
*C. henrici*	15	−0.4548 *	19	−0.4960 *
*C. irus* (1992–2013)	22	−0.6507 **	--	
1994–2013	20	−0.5985 **	--	

**Table 6:** Spearman rank correlation coefficients (r) of first observed date for *C. irus* with the other study species’ first observed date, calculating dates either using surveys only in central Wisconsin or using all survey data including from northern Wisconsin and northeastern Minnesota. * *p* < 0.05, ** *p* < 0.01, *** *p* < 0.001.

Species	First observed date central Wisconsin only		All data, including further north	
	N	r	N	r
*C. augustinus*	19	+0.5457 *	20	+0.5269 *
*C. polios*	20	+0.7753 ***	20	+0.5980 **
*C. niphon*	20	+0.7564 ***	20	+0.7529 ***
*C. henrici*	15	+0.8105 **	19	+0.5617 *

**Table 7:** Spearman rank correlation coefficients (r) of trend over time (correlations with year) in consecutive-year change in first observed date (absolute value N days change between the prior year’s and the current year’s first observed date), by species. Dates are calculated both using surveys only in central Wisconsin and using all survey data including from northern Wisconsin and northeastern Minnesota. + *p* < 0.10, * *p* < 0.05, ** *p* < 0.01, *** *p* < 0.001.

Species	First observed date, central Wisconsin only		All data for date, including further north	
	N	r	N	r
*C. augustinus*	17	+0.3106	19	+0.4875 *
*C. polios*	19	+0.1936	19	+0.3357
*C. niphon*	19	+0.3322	19	+0.4187 +
*C. henrici*	12	+0.8056 **	17	+0.5479 *
*C. irus* 1992–2013	21	+0.4795 *	21	+0.4795 *

All correlations of FOD and temperature were negative (Table 8), significantly so for all species except *C. augustinus*. Each elfin species’ abundance correlated negatively with FOD that year, significantly so for all species except *C. polios* (Table 9). Nearly uniformly, each elfin species’ abundance correlated negatively with last year’s FOD, significantly so except for *C. irus* (Table 9). For all five species, flight period length strongly and significantly co-varied with number of individuals recorded that year (Table 10). Total *C. irus* individuals per total survey time in flight period each year significantly negatively correlated with first observed date (Spearman rank correlation r = −0.49519, N = 22 years during 1992–2013, *p* < 0.05). This included all sites surveyed in *C. irus* range and timing each year, not just the long-term sites. 

**Table 8:** Spearman rank correlation coefficients (r) of first observed date (FOD), calculated using either surveys only in central Wisconsin (central) or all survey data including from northern Wisconsin and northeastern Minnesota (all), with spring temperature (March-April-May) through 2011 in the west central or central Wisconsin regions. + *p* < 0.10, * *p* <0.05, ** *p* < 0.01.

Species	Spring temperature, west central				Spring temperature, central			
	FOD central		FOD all		FOD central		FOD all	
	N	r	N	r	N	r	N	r
*C. augustinus*	16	−0.0458	18	−0.1274	16	−0.0739	18	−0.1668
*C. polios*	18	−0.4758 *	18	−0.4956 *	18	−0.5565 *	18	−0.5953 **
*C. henrici*	13	−0.5125 +	17	−0.5354 *	13	−0.6455 *	17	−0.6373 **
*C. niphon*	18	−0.5539 *	18	−0.6047 **	18	−0.5899 *	18	−0.6594 **
*C. irus*	20	−0.6147 **	--		20	−0.6584 **	--	

**Table 9:** Spearman rank correlation coefficients (r) of abundance (individuals per hour on peak count per site per year) and first observed date (FOD), calculated either using surveys only in central Wisconsin or using all surveys including from northern Wisconsin and northeastern Minnesota. * *p* < 0.05, ** *p* < 0.01, *** *p* < 0.001.

Species	FOD, central only		FOD, all data including farther north	
	N	r	N	r
Current year abundance				
*C. augustinus* (all)	288	−0.1984 **	320	−0.1795 **
more reliable sites ^1^	90	−0.3284 **	100	−0.2956 **
less reliable sites ^2^	198	−0.1616 **	220	−0.1425 *
*C. polios*	180	−0.0765	180	−0.0447
*C. niphon*	300	−0.0669	300	−0.1159 **
*C. henrici*	165	−0.1641 *	209	−0.2760 ***
*C. irus* (all)	358	−0.0671	NA	
extant sites ^3^	170	−0.2251 **	NA	
extant years ^4^	251	−0.1908 **	NA	
Next year’s abundance				
*C. augustinus* (all)	272	−0.1012	304	−0.1334
more reliable sites ^1^	85	−0.2368 *	95	−0.2469 *
less reliable sites ^2^	187	−0.0432	209	−0.0431
*C. polios*	171	−0.1909 *	171	−0.1187
*C. niphon*	285	−0.1805 **	285	−0.1933 **
*C. henrici*	154	−0.0526	198	−0.1830 **
*C. irus* (all)	341	+0.0299	NA	
extant sites ^3^	162	−0.0692	NA	
extant years ^4^	243	−0.0714	NA	

^1^ recorded present >5 years out of 20. ^2^ recorded present 1–3 years out of 20. ^3^ limited to sites where we recorded the species as present in 2012 and/or 2013. ^4^ limited time series to the last year we recorded the species as present plus one more year.

**Table 10:** Spearman rank correlation coefficients (r) of N days in flight span per year and N individuals recorded at all sites (not just long-term sites) on all surveys in central Wisconsin that year, by species. * *p* < 0.05, ** *p* < 0.01, *** *p* < 0.001.

Species	N	r
*C. augustinus*	20	+0.8955 ***
*C. polios*	20	+0.7896 ***
*C. henrici*	20	+0.9125 ***
*C. niphon*	20	+0.6049 **
*C. irus* 1992–2013	22	+0.5241 *
1994–2013	20	+0.4642 *

### 3.2. Climate and Elfin Abundance

*C. irus* showed climate influences only on the current year’s abundance and *C. polios* only on the next year’s abundance (Table 11). The other elfins showed climate influences on both the current year’s and next year’s abundance. Most correlations with temperature were positive. Although *C. augustinus* positively correlated with cooler winters, *C. irus* co-varied with warmer springs, and *C. augustinus*, *C. niphon* and *C. henrici* with the previous year’s warmer spring and growing season (*C. niphon* only). Relationships to precipitation were usually positive. Although *C. irus* and *C. augustinus* correlated negatively with season-long snowfall, *C. henrici* correlated positively with wet winters. *C. niphon* co-varied with current spring precipitation, and all species except *C. irus* with last year’s summer, growing season, and/or annual precipitation. However, as a lag effect, *C. henrici* correlated negatively with the prior spring’s precipitation. 

### 3.3. Variation among Years

**Figure 3:**
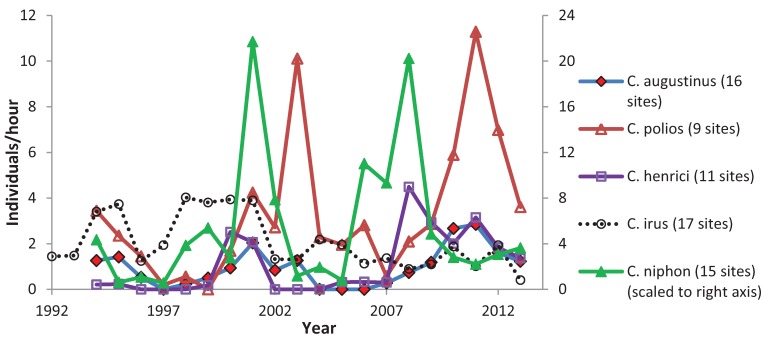
Mean abundance on peak count per year at the study sites in central Wisconsin, by species.

**Figure 4:**
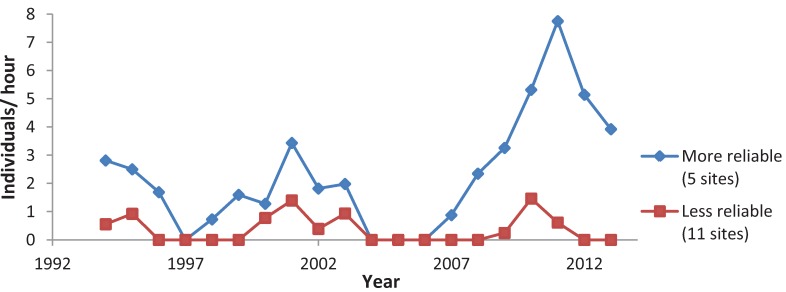
Mean *C. augustinus* abundance, for more reliable sites (recorded present >5 years out of 20) and less reliable sites (recorded present only 1–3 years out of 20).

**Figure 5:**
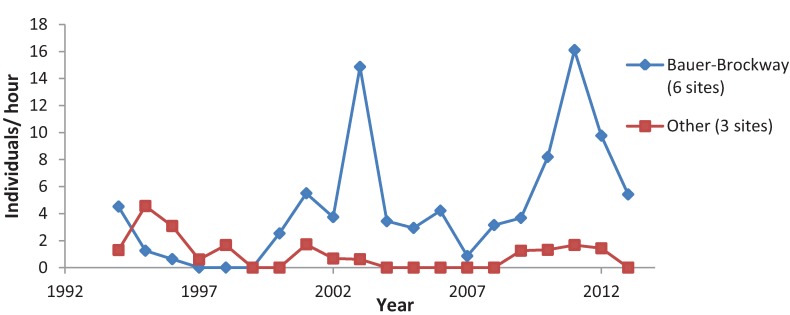
Mean *C. polios* abundance in the Bauer-Brockway cluster of sites compared to the other three long-term sites.

**Figure 6:**
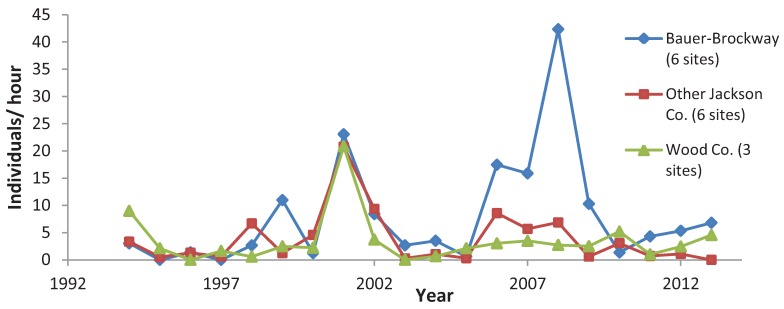
Mean *C. niphon* abundance by three site groupings.

**Figure 7:**
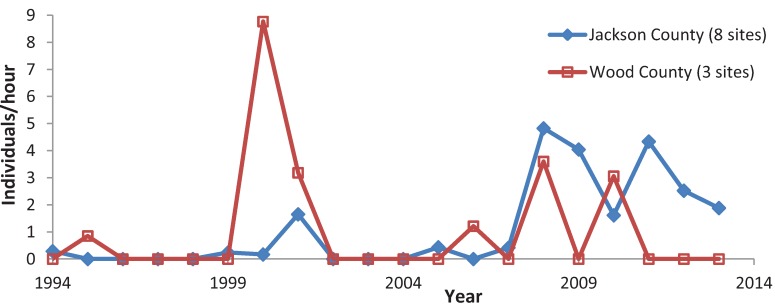
Mean *C. henrici* abundance, by county.

**Figure 8:**
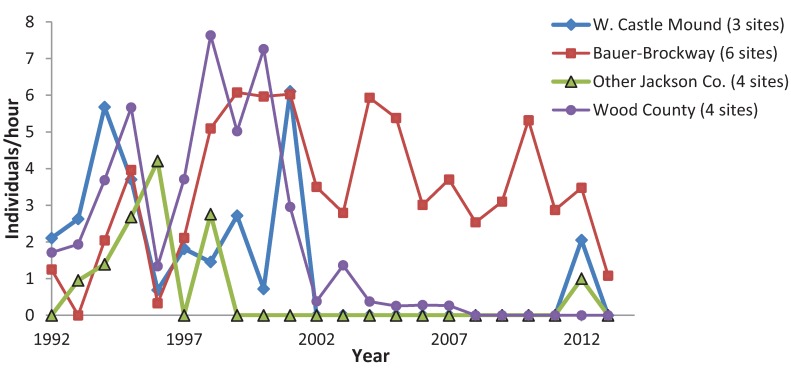
Mean *C. irus* abundance, by geographical groupings.

**Figure 9:**
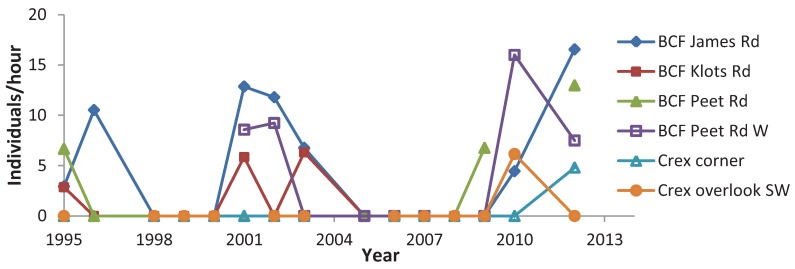
*C. henrici* abundance in northwestern Wisconsin sites (Figure 1) in Burnett County Forest (BCF) and Crex Meadows. Spearman rank correlation of trend (abundance *versus* year): r = +0.12196, N = 76 abundance indices from six sites surveyed nine to fifteen times during 1995–2012.

When testing all sites together, four species had positive population trends: *C. polios* and *C. henrici* showed a significant increase (Table 12) whereas *C. irus* showed a significant decrease. Trends in reserves tended to be more positive than in non-reserves (Table 12). *C. irus* significantly decreased in both types of non-reserve sites, but had a non-significant negative trend in reserves. Of the five elfins, *C. irus* had, however, the most negative trend in reserves. *C. henrici* and *C. niphon* significantly increased in reserves. *C. niphon* did not show any significant trends in non-reserves; *C. henrici* had weaker positive trends in non-reserves, although significant in shifting mosaic. *C. polios* and *C. augustinus* did not have any significant patterns by site type. The mean abundance for each elfin species at the monitoring sites varied greatly among years (Figure 3). Most species were at a low point in 1997 and 2004. Differences in trend were also evident when subdividing the long-term survey sites geographically (Figure 4, Figure 5, Figure 6, Figure 7 and Figure 8). While *C. henrici* significantly increased in central Wisconsin during the study period (Table 12), this pattern was not as strong in the smaller dataset from northwestern Wisconsin (Figure 9), where the trend over time was positive but not significant. *C. irus* declined most in Wood County (Figure 8), where we recorded no *C. irus* in any of the sites after 2007, including reserves (Table 2). 

**Table 11:** Results of Spearman rank correlations of abundance of each study species each year in each study site with climate factors and first observed date (FOD) (Table 9) of that species, by species. The conditions significantly correlated with higher abundance are described. “Flood” = significantly correlated with soil moisture at the high end of the spectrum on the Palmer Index (see Methods). See Appendix Table A1 for numerical test results.

Species	Current year abundance			Next year’s abundance				
Winter	Winter	Spring	FOD	Spring	Summer	Growing Season	Year	FOD
*C. augustinus* (all)	Cool		earlier	warm	wet	wet	wet	
					flood	flood		
more reliable ^1^	Cool		earlier		wet	wet	wet	earlier
					flood	flood		
less reliable ^2^	<snow		earlier		wet	wet		
*C. polios*					flood	wet		earlier
						flood		
*C. niphon*		wet	earlier	warm		warm	warm	earlier
		flood					flood	
*C. henrici*			earlier	warm			flood	earlier
	Flood			dry				
*C. irus* (all)		warm						
extant sites ^3^	<snow	warm	earlier					
extant years ^4^	<snow	warm	earlier					

^1^ sites where we recorded the species present >5 years out of 20. ^2^ sites where we recorded the species present only 1–3 years out of 20. ^3^ limited to sites where we recorded the species as present in 2012 and/or 2013. ^4^ limited time series to the last year we recorded the species as present plus one more year.

**Table 12:** Spearman rank correlation coefficients (r) of abundance (individuals per hour on peak count per site per year) and year (trend over time), by species and site type. + *p* < 0.055, * *p* < 0.05, ** *p* < 0.01, *** *p* < 0.001.

Species ^1^		Reserves		Permanency of Habitat		Shifting Mosaic		All sites	
		N	r	N	r	N	r	N	R
N	*C. augustinus*	120	−0.0070	100	+0.0826	100	+0.0436	320	+0.0326
N	*C. polios*	80	+0.2160 +	20	+0.1782	80	+0.1315	180	+0.1706 *
S	*C. niphon*	80	+0.2310 *	80	−0.0184	140	+0.0929	300	+0.0923
S	*C. henrici*	100	+0.3306 **	60	+0.1022	60	+0.2686 *	220	+0.2590 ***
S	*C. irus* all	126	−0.0534	84	−0.4903 ***	148	−0.2002 *	358	−0.2056 ***
	1994–2013	120	−0.1057	80	−0.4822 ***	140	−0.2419 **	340	−0.2413 ***

^1^ N = northern species, S = southern species.

## 4. Discussion

### 4.1. First Observed Date (FOD)

FODs for most elfins were significantly earlier when spring was warmer (Table 8, except *C. augustinus*), as was expected from the literature correlating variation in butterfly flight periods with variation among years in seasonal development [32,33,34,35]. It was also unsurprising that FOD became earlier during the study (Table 3, Figure 2), as already reported in recent decades for elfins, other butterflies, and many other taxa [34,35,36,37]. Winter and spring 2012 were the warmest on record [38], and most Wisconsin spring butterflies in a volunteer dataset had their record earliest dates in 2010, until most of these records were broken in 2012 [39]. 

However, we did not anticipate that earlier phenologies would correspond to dramatically longer flight periods (Table 5, except *C. polios*) as well as higher elfin numbers (Table 10) and relative abundances, both as a current-year relationship (Table 9, except *C. polios*) and as a lag-year effect (Table 9, except for *C. irus*). This analysis cannot distinguish whether the lag effect is explained by climatic patterns or by the longer flight period associated with earlier FODs. It is also unclear whether the recently developing pattern of significantly greater variation in FOD between consecutive years (Table 7) will persist. 

Although FOD can have some biases due to variation in sites and sampling intensity among years [40], our study is less prone to this because sampling effort and sites were relatively similar among years, and observers remained constant throughout the study. The strong negative correlations of FOD with spring temperature (Table 8) validate these dates. But the strong correlations of FOD with elfin abundance also support that FOD corresponds both to phenology and butterfly abundance [40]. 

### 4.2. Climate and Elfin Abundance

The apparent climatic effects on elfin abundance are consistent with numerous studies that link climatic variation to changes in butterfly abundance [1,2,41,42,43,44]. Many of these climatic relationships (Table 11) were logical in relation to the known range of the study species [20,21,22]. In Wisconsin, *C. irus* and *C. henrici* are at the north (cool) and west (dry) end [24,45] of their known ranges. These species co-varied with warmer springs, and *C. henrici* with moister winters. However, *C. irus* increased with lower season-long snowfalls and as a lag, *C. henrici* increased with drier springs. *C. niphon* also ranges widely south and east of the study area, and its significant relationships to climate were positive with warmth and moistness. Conversely, *C. augustinus* is at the southern end of its range in the Midwest, although it occurs well south of Wisconsin farther east in Atlantic coastal states, and *C. polios* has the most northerly range of the study species. *C. augustinus* had the only significant negative relationship to temperature (preferring cooler winters), although it also co-varied with spring warmth as a lag effect. Otherwise, the two most northern species responded to precipitation, with *C. augustinus* correlating negatively with season-long snowfall but both species preferring moister summers and growing seasons. For all species, we recorded more individuals (Table 10) and higher relative abundances either as a current-year or lag-year effect (Table 9) with earlier first observed dates. Thus, it appears that southern elfins preferred warmth and northern elfins were not directly limited by warmth. In other words, these species appear to tolerate climatic conditions outside their observed geographic ranges. Consistent with this, Warren *et al.* [43] noted that the ranges of many British butterfly species appeared limited by factors other than climate, since they were not occupying all areas that were climatically suitable. Likewise, both expected and surprising changes in butterfly fauna occurred in a re-survey of a Swedish Arctic alpine national park compared to 60 years ago [46]. Butterfly species richness increased, as expected, with northward or uphill expansion of southern species [47,48]. However, the downhill expansion in the range of high alpine butterflies was surprising, to the point that no net uphill shift in butterfly ranges was evident [46]. These unexpected outcomes may result from the complexity of seasonal variation in both temperature and precipitation. Such positive responses of northern species would be temporary if continued climate change resulted in vegetative shifts unsuitable for these butterflies [46,49]. 

### 4.3. Variation among Years

Given the positive response of all elfin study species to earlier phenology (Table 9 and Table 10), and significantly earlier trend in FOD during the study (Table 3), it is not surprising that overall trends for most species were positive (Table 12). The exception was the specialist *C. irus*, which decreased significantly despite positive relationships to warm springs and earlier phenologies. Furthermore, only one of three southern species (*C. henrici*) had a significant positive trend in all sites combined, yet one of two northern species (*C. polios*) did. However, for four of these species, overall trends were positive (regardless of significance). 

By comparison, Breed *et al.* [44] calculated a positive trend for the same five elfin species. In their analysis, the same two elfins categorized as northern species (*C. augustinus*, *C. polios*) had the mildest increases, and *C. irus* had the strongest increase. When geographically partitioned (supplemental material in [44]), lupine-feeding populations of *C. irus* in interior sites appeared to decline while *Baptisia*-feeding populations in coastal sites, where active conservation habitat management had been reported [8,9], increased. A positive outcome for lupine-feeding *C. irus* has also followed conservation management designed and implemented for this species [10,11]. Likewise, in our dataset, trends in reserves tended to be more positive than in non-reserves (Table 12). *C. irus* showed the most benefit of reserves (*i.e.*, the biggest increase in trend in reserves compared to non-reserves), which were managed under regulations for *L. melissa samuelis*, which shares the same host plant. However, of the five elfins, *C. irus* also had the most negative trend in reserves. 

Caution should be used in any application of these analyses to predict future elfin responses to climatic variation. Higher abundance during some years may be partly a result of the greater amount of time spent flying by butterflies in warm springs [50] making them more apparent, thus increasing our counts. It might also be possible for butterflies to shift their phenology in response to climate change (an effect we appear to have observed) without climate change negatively affecting their population, if there is adequate habitat for them [51]. We employed one analytical method (correlations), while climatic variables could have other effects as well, such as threshold and non-linear impacts. In addition, these populations appear to have a lag effect of abundance relative to the same climatic conditions. For example, *C. henrici* abundance in 2013 (Figure 3) was higher than in 1994 and 2003, which had similar first observed dates (Figure 2). Furthermore, it may take more surveys per site in a year than we did to detect all elfin populations actually present each year, because detection probabilities may be low for these species [13]. Thus, some populations were likely present in some years in which our population abundance index was zero. We are treating zero here as a relative abundance and are not statistically distinguishing that low abundance from true absence. The large variation in annual abundance of elfins in this 20–22-year study and the uncertainty about longer-term effects of climate highlight the value of even longer monitoring periods than this [4]. 

## 5. Conclusions

It is difficult but important to disentangle climatic and landscape influences on butterfly population abundance and trend [52]. The strong consistent relationship of elfin abundance to phenology suggests that climate is a contributor to the positive elfin abundance patterns found here. On the other hand, suitable climate appears insufficient to counterbalance unsuitable vegetative and landscape trends, as reported also by others [43,48,53]. This is evidenced by the decline of *C. irus*, even though the recent pattern of warmer springs and earlier phenologies appear positive for *C. irus* abundance. Fortunately, conservation measures for a suitable umbrella species appear to be ameliorating the overall negative *C. irus* trend. This underscores how essential reserves are for providing consistent suitable vegetative resources and conditions so that butterflies can find the resources and buffers necessary to take advantage of or withstand climatic variation [54]. However, conservation programs for *L. melissa samuelis* may not be providing sufficient benefit for *C. irus*, which has narrower vegetative and management tolerances [17]. Thus, monitoring programs and habitat management specifically for *C. irus* [8,9,10,11,13] appear necessary to obtain a long-term stable trend for this species in Wisconsin.

## Appendix

**Table A1:** Significant Spearman rank correlations of elfin abundance and climate variables, as summarized in Table 11. “Palmer” = Palmer Drought Severity Index (see Methods). * *p* < 0.05, ** *p* < 0.01, *** *p* < 0.001.

Species and climatic factor	Current year abundance		Next year’s abundance	
*C. augustinus* all sites				
Winter temperature	272	−0.1513 *		
Spring temperature			288	+0.1271 *
Summer Palmer			272	+0.1436 *
Summer precipitation			288	+0.2267 ***
Growing season Palmer			272	+0.1619 *
Growing season precipitation			288	+0.2033 ***
Annual precipitation			288	+0.1565 *
*C. augustinus* more reliable sites				
Winter temperature	85	−0.2346 *		
Summer Palmer			85	+0.2220 *
Summer precipitation			90	+0.3430 **
Growing season Palmer			90	+0.2224 *
Growing season precipitation			90	+0.2748 **
Annual precipitation			90	+0.2167 *
*C. augustinus* less reliable sites				
Season-long snowfall	187	−0.1500 *		
Summer precipitation			198	+0.1675 *
Growing season precipitation			198	+0.1653 *
*C. polios*				
Summer Palmer			153	+0.1853 *
Growing season Palmer			153	+0.1724 *
Growing season precipitation			162	+0.2010 *
*C. henrici*				
Winter Palmer	176	+0.1616 *		
Spring Palmer			187	−0.1808 *
Spring temperature			198	+0.2086 **
*C. niphon*				
Spring Palmer	255	+0.1836 **		
Spring precipitation	270	+0.2129 **		
Spring temperature			270	+0.2298 ***
Growing season temperature			270	+0.2361 ***
Annual temperature			270	+0.2123 ***
Annual Palmer			240	+0.1426 *
*C. irus* all				
Spring temperature	324	+0.1183 *		
*C. irus* extant sites				
Season-long snowfall	146	−0.1949 *		
Spring temperature	154	+0.2148 **		
*C. irus* extant years				
Season-long snowfall	227	−0.1820 **		
Spring temperature	235	+0.2030 **		
